# Analyzing Mushroom Structural Patterns of a Highly Compressible and Expandable Hemostatic Foam for Gastric Perforation Repair

**DOI:** 10.1002/advs.202306917

**Published:** 2024-03-04

**Authors:** Zhenzhen Shu, En Liu, Yu Huang, Qiang Luo, Tongchuan Wang, Xin Li, Kibret Mequanint, Shiming Yang, Malcolm Xing, Chaoqiang Fan

**Affiliations:** ^1^ Department of Gastroenterology Xinqiao Hospital Army Medical University NO.183, Xinqiao Street Chongqing 400037 China; ^2^ Department of Chemical and Biochemical Engineering and School of Biomedical Engineering The University of Western Ontario London Ontario N6A 5B9 Canada; ^3^ Chongqing Municipality Clinical Research Center for Gastroenterology Chongqing 400037 China; ^4^ Department of Mechanical Engineering University of Manitoba Winnipeg Manitoba R3T 2N2 Canada

**Keywords:** asymmetric hemostasis, gastric perforation, golden ratio in layered structure, gradient porous structure, mathematic modeling for 3D printing, super‐compressive elasticity

## Abstract

Nature presents the most beautiful patterns through evolving. Here, a layered porous pattern in golden ratio (0.618) is reported from a type of mushroom ‐*Dictyophora Rubrovalvata* stipe (DRS). The hierarchical structure shows a mathematical correlation with the golden ratio. This unique structure leads to superior mechanical properties. The gradient porous structure from outside to innermost endows it with asymmetrical hydrophilicity. A mathematical model is then developed to predict and apply to 3D printed structures. The mushroom is then explored to repair gastric perforation because the stomach is a continuous peristaltic organ, and the perforated site is subject to repeated mechanical movements and pressure changes. At present, endoscopic clipping is ineffective in treating ulcerative perforation with fragile surrounding tissues. Although endoscopic implant occlusion provides a new direction for the treatment of gastric ulcers, but the metal or plastic occluder needs to be removed, requiring a second intervention. Decellularized DRS (DDRS) is found with asymmetric water absorption rate, super‐compressive elasticity, shape memory, and biocompatibility, making it a suitable occluder for the gastric perforation. The efficacy in blocking gastric perforation and promoting healing is confirmed by endoscopic observation and tissue analysis during a 2‐month study.

## Graphical abstract

A type of mushroom: structural analysis, modeling, super compressive‐expansive mechanics, hemostasis, and its potential strategy for porcine gastric perforation.

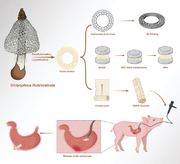

## 1 Introduction

Gastric perforation is a disease of the stomach cavity and abdominal cavity caused by damage to the stomach wall. The main cause of acute gastric perforation is severe gastric ulcer.^[^ ^1^ ^]^ Gastric perforation results in the flow of chemically stimulated gastrointestinal contents into the abdominal cavity, which can cause severe abdominal pain and acute diffuse peritonitis.^[^ ^2^ ^]^ In addition, gastric perforation is often accompanied with bleeding, and it is difficult to stop bleeding in acidic gastric juice.^[^ ^3^ ^]^ Emergency treatment is needed, otherwise, it may cause inflammatory reactions and even endanger the life.^[^ ^4^ ^]^ Surgical repair has long been the standard of care for acute gastric perforation. However, surgical treatment of perforation is relatively invasive, operation time is long, and treatment may be delayed.^[^ ^5^ ^]^ Endoscopic titanium clip closure of perforation is also widely used in the clinic, but for ulcerative perforation with rigid surrounding tissue, the treatment effect of titanium clip is not good.^[^ ^6^ ^]^ Endoscopic implant plugging provides a promising approach to address gastric perforation. However, most current plugging materials are nondegradable metal or plastic and require a secondary surgery for removal.^[^ ^7^, ^8^ ^]^ Therefore, it is important to develop a new occluder with biodegradable, excellent biocompatibility, superior mechanical properties, local hemostasis, and endoscopic delivery for the treatment of gastric perforation.

Nature always finds her way to harmonize their evolving with environments, which generates new composition of materials and their novel assembled array in morphology.^[^ ^9^, ^10^, ^11^, ^12^, ^13^ ^]^ By studying the structure, function and organizational principle of organisms, humans have invented technologies with superior performance than otherwise possible.^[^ ^14^, ^15^, ^16^, ^17^, ^18^ ^]^ However, the use of natural materials for the treatment of gastric perforation has not been reported. *Dictyophora rubrovalvata* (DR), an edible mushroom in the *Dictyophora* genus, which has been widely reported for its anti‐inflammatory, antioxidant and anti‐tumor medicinal values.^[^ ^19^, ^20^, ^21^ ^]^ The stipe of DR (DRS) has a special porous structure, but there are few detailed analyses of the structure of DRS. In our preliminary findings, the cross‐section of DRS has a gradient porous structure, and the ratio of pore diameter of each layer is close to the golden ratio (0.618).^[^ ^22^, ^23^, ^24^, ^25^ ^]^ This magical structure is related to the special growth mode of DR. On the day of maturity, the DR breaks through its shell and can quickly elongate from 3–4 to 15 cm even 20 cm within a few hours. Compared to solid structures, porous hollow structures are more suitable for this rapid elongation and expansion process. In addition, the outside of the DRS has reticular open pores, while the inside doesnot have. They may play a crucial role in water regulation, allowing for the uptake and release of water as needed by the fungus. This change leads to different porosities between inside and outside surfaces, which in turn affect the wetting properties and hydrophilicity. In fact, recent studies have confirmed that the hydrophobics can enhance hemostatic effects.^[^ ^26^, ^27^ ^]^

We hypothesized that these features may be suitable for repairing dynamic tissues that experience pressure and mechanical movement, such as a gastric perforation.^[^ ^28^, ^29^ ^]^ At present, the application potential of DR has not been fully developed. By chemical acellular and freeze‐drying methods, we obtained decellularized DRS (DDRS).^[^ ^30^, ^31^, ^32^ ^]^ To examine the performance of DDRS, we analyzed the structure of natural DRS in detail, expressed its mathematical rules through mathematical formulas, and constructed the bionic scaffold of DRS Through computer simulation 3D printing. And then, we studied the degradation, biocompatibility, mechanical properties, and hemostatic properties of DDRS. Finally, we blocked the porcine gastric perforation model by delivering DDRS under endoscope.

## 2 Results and Discussion

### 2.1 Preparation and Morphological Characterization of DDRS

**Figure 1:**
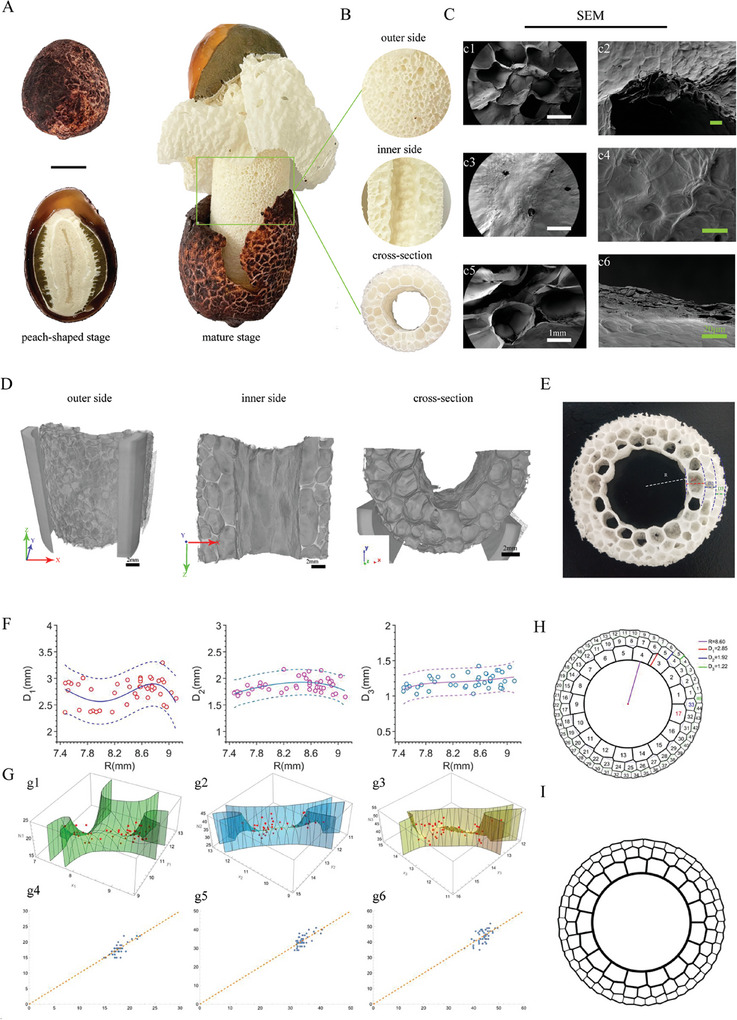
Morphology and structure of DRS. A) The developmental process of *Dictyophora rubrovalvata* (DR). Left: peach‐shaped stage, right: mature stage. B) Enlarged view of the inner side, outer side and cross‐section of DRS. C) SEM images: (c1,c2) outer side, (c3,c4) inner side, (c5,c6) cross‐section at different magnifications. D) Micro‐CT images of the structure in different directions. E) Porous structure, R represents the cell radius, and D_1_, D_2_, viewand D_3_ represent the average inner diameter of each layer, respectively (Video S1, Supporting Information). F) Linear chart representing the relationship between R and D_1_, D_2_, and D_3_. G) Set the number of polygons in each layer to N_1_, N_2_, and N_3_, respectively. 2D curves representing the relationship between D, R and N (x_1_ = R, y_1_ = R+D_1_, x_2_ = R+D_1_, y_2_ = R+D_1_+D_2_, x_3_ = R+D_1_+D_2_, y_3_ = R+D_1_+D_2_+D_3_) (g1,g2,g3). Predictive calibration charts represent the relationship between the actual predicted value and the true value of N (g4,g5,g6). H,I) Computer simulated cross‐section image and the callout drawings of DRS.

DR is an edible fungus. The development process from peach shaped stage to the mature stage only a few hours (**Figure** **1**A). Due to this special growth mode, the stipe of DR (DRS) has asymmetrical structures on three sides (Figure 1B). The outside of DRS has semi‐opening porous structures (opening cells) that are directly exposed to the growth environment (Figure 1C(c1,c2)). However, interestingly, the inner surface is a smooth membrane covering close cells (pores) (Figure 1C(c3,c4)). The stratified porous structure was observed in the cross section (Figure 1C(c5,c6)). The Micro‐CT images show that the cell diameter decreases gradually from inside to outside (Figure 1D, Video S1, Supporting Information). The structure with a special internal arrangement is a unique and fascinating feature of this type of fungus.

We found that the pores generate regularly, particularly in the cross‐section. The interior is hollow and almost circular, and polygonal pores naturally separate the substantial part into three layers. We define the radius of the hollow section as R, and the average diameter of each layer from the inside to the outside is D_1_, D_2_, D_3_ (Figure 1E). The relationship model between R and D shows a significant composite quartic linear model (Figure 1F). Through regression analysis, we found the following equations:

(1) $$\begin{array}{ccc} D_{1} & = & -3594.43+1818.29\times R-343.56\times R^{2}\\ & & +\;28.76\times R^{3}-0.90\times R^{4} \end{array}$$

(2) $$\begin{array}{ccc} D_{2} & = & -283.03+139.84\times R-25.99\times R^{2}\\ & & +\;2.16\times R^{3}-0.07\times R^{4} \end{array}$$

(3) $$\begin{array}{ccc} D_{3} & = & -512.63+241.18\times R-42.40\times R^{2}\\ & & +\;3.31\times R^{3}-0.10\times R^{4} \end{array}$$

These equations make it possible to predict the size of the three inner diameters (D_1_, D_2_, D_3_) as a function of the size of the hollow radius R. We believe that the two radii of each layer circle determine the number of polygons inside and fix the number of polygons in three layers (N_1_, N_2_, N_3_). Therefore, we have constructed three binary complete polynomial regression models to predict the number of each layer accurately:

(4) $$\begin{array}{ccc} N_{1} & = & \sum_{i=0}^{n}\sum_{j=0}^{n}\beta_{\mathrm{ij}}x_{1}^{i}y_{1}^{j}(x_{1}=R,y_{1}=R+D_{1},\\ & & \beta \mathrm{isth}\mathrm{emod}\mathrm{elfi}\mathrm{ttin}\mathrm{gcoe}\mathrm{ffic}\mathrm{ient}) \end{array}$$

(5) $$\begin{array}{ccc} N_{2} & = & \sum_{i=0}^{n}\sum_{j=0}^{n}\gamma_{\mathrm{ij}}x_{2}^{i}y_{2}^{j}(x_{2}=R+D_{1},\\ & & y_{2}=R+D_{1}+D_{2,}\gamma \mathrm{isth}\mathrm{emod}\mathrm{elfi}\mathrm{ttin}\mathrm{gcoe}\mathrm{ffic}\mathrm{ient}) \end{array}$$

(6) $$\begin{array}{ccc} N_{3} & = & \sum_{i=0}^{n}\sum_{j=0}^{n}\delta_{\mathrm{ij}}x_{3}^{i}y_{3}^{j}(x_{3}=R+D_{1}+D_{2,}y_{3}=R+D_{1}+D_{2}\\ & & +\;D_{3},\delta \mathrm{isth}\mathrm{emod}\mathrm{elfi}\mathrm{ttin}\mathrm{gcoe}\mathrm{ffic}\mathrm{ient}) \end{array}$$

Through analysis using MATLAB software, we found that when the degree of n is 3, it can achieve a better prediction effect. From the 2D model diagram representing the relationship between D, R, and N, it can be observed that there is an excellent curvilinear relationship between them. The predictive calibration diagram shows that the predicted value is close to the actual value (Figure 1G). By setting the hollow radius R of DRS, using the previous model to predict D_1_, D_2_, D_3_, N_1_, N_2_, N_3_, and simulate the section mode diagram of DRS by computer simulation. For example, when we take the hollow radius R = 8.6 (the median of the actual measured hollow radius), we can calculate the following parameters through the previous model:

(7) $$\begin{array}{c} D_{1}=2.85,D_{2}=1.92,D_{3}=1.22,N_{1}=17,N_{2}=33,N_{3}=45 \end{array}$$

Using the above seven parameters and MATLAB software, we finally obtained the computer‐simulated images and annotation drawings of the cross‐section of DRS (Figure 1H,I).

### 2.2 3D Printed Silicone Model and their Compression Elasticity

**Figure 2:**
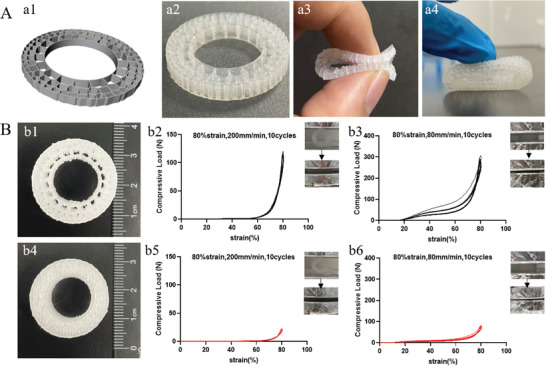
3D simulated model. A) Simulated DRS model (a1), Biomimetic DRS model obtained by biological silicone printing (a2) (Video S2, Supporting Information), which can be folded (a3) and compressed (a4). B) A simulated DRS model. (b1) printed by biological silicone (2.5 cm in height, 8 mm in thickness), (b2) The stress–strain curve of the DRS model under cyclic compression in the direction parallel to the transverse plane, and images in compression experiments (Video S3, Supporting Information), (b3) The stress–strain curve of the DRS model under cyclic compression in the direction perpendicular to the transverse plane, and images in compression experiments. A concentric cylindrical model(Video S3, Supporting Information), (b4) printed by biological silicone (2.5 cm in height, 8 mm in thickness), (b5) The stress–strain curve of the concentric circle model under cyclic compression in the direction parallel to the transverse plane, and images in compression experiments, (b6) The stress–strain curve of the concentric circle model under cyclic compression in the direction perpendicular to the transverse plane, and images in compression experiments(n = 3, *p* <0.05).

Through computer modeling, the 3D printed object (**Figure** **2**A(a1),) of the DRS model was successfully obtained with the biomimetic elastics (Figure 2A(a2); Video S2, Supporting Information). It can be folded and compressed using silicone (Figure 2A(a3,a4)). A DRS model (2.5 cm in diameter and 8 mm thick) was used for compression experiments, compare with a concentric circle model of the same size (Figure 2B(b1,b4)). The maximum compressive strength (Figure 2B(b2,b3); Video S3, Supporting Information) of the model is significantly greater than that of the concentric circle model (Figure 2B(b5,b6)) in both parallel and vertical directions.

### 2.3 Morphological Analysis of DDRS

**Figure 3:**
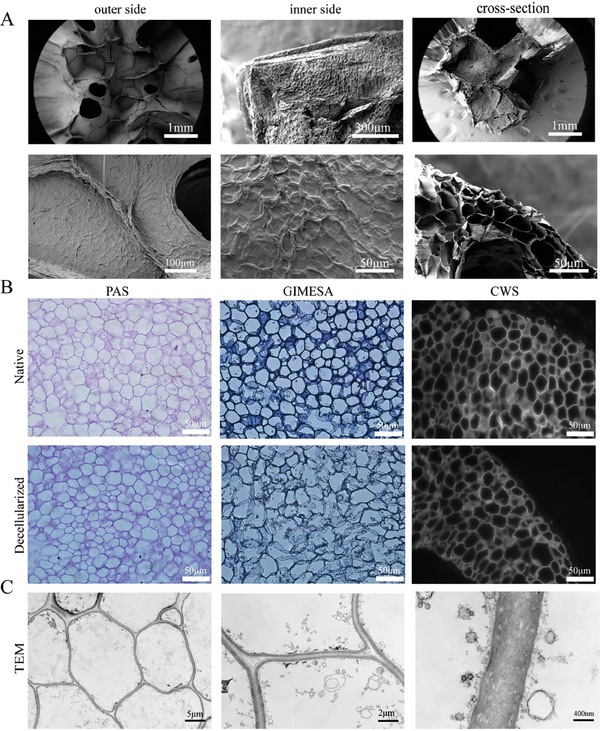
Morphology and decellularization effect of DDRS. A) SEM images of the outer, inner, and cross‐section of DDRS. B) Images of different staining methods to observe the paraffin sections of DRS before and after acellularization (PAS, Giemsa, CWS). C) TEM images of the cross‐section of DDRS.

After decellularization, we investigated the DDS morphology using SEM (**Figure** **3**A). It is noticeable that the macro and micro‐structures of DDRS are almost the same with DR (Video S4, Supporting Information). Proved that the appearance of DR did not change significantly after the decellularized lyophilization treatment. To evaluate the decellularized effect, different coloring methods were used to observe DRS paraffin sections before and after treatment (Figure 3B). It is evident that, under different coloring methods, there was no significant change. Nuclei or cytoplasm were not detected. For the ultra‐structure of the DDRS, TEM analysis (Figure 3C) demonstrated the absence of nuclear or cytoplasmic structure in the cell. An apparent boundary between the cellular walls forms a dark‐bright‐dark system, which may facilitate water molecules’ transportation.

### 2.4 Water Absorption, Permeability, and Mechanical Properties of DDRS

**Figure 4:**
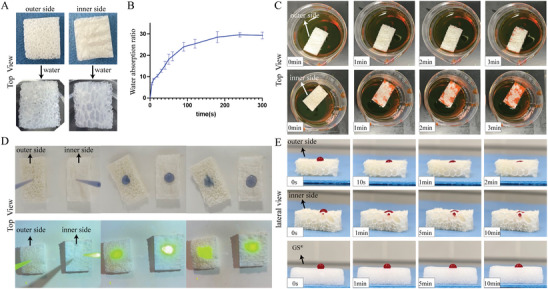
Water absorption of materials and different permeability of inner side and outer side of DDRS. A) Images on the inner and outer sides of DDRS before and after water absorption. B) Water absorption of DDRS at different time points. C) Permeability of inner and outer sides of DDRS at different times. The red liquid represents the diluted magenta solution (Video S5, Supporting Information). D) Permeability of inner and outer sides of DDRS at different times. The blue liquid represents diluted Giemsa solution, and the yellow liquid is diluted sodium fluorescein (Videos S6 and S7, Supporting Information). E) Blood permeability of inner and outer sides DDRS at different times (Videos S8–S10, Supporting Information).

Water absorption can reach over 2000% (**Figure** **4**A,B), due to the porous structure and composition of polysaccharides and chitin.^[^ ^33^ ^]^ However, there is the difference between the inner and outer sides, which have disparity absorption of liquids.^[^ ^34^, ^35^, ^36^, ^37^ ^]^ The outer side reached a faster permeation rate (Figure 4C; Video S5, Supporting Information). When Giemsa and sodium fluorescein solutions were dripped DDRS, the penetration rate from the outer side was faster than the other side (Figure 4D; Videos S6 and S7, Supporting Information). Blood (sodium citrate) were also added to the surface with similar results. In addition, the liquid on the inside surface has a temporary residence identical to that of the gelatin sponge (GS) and then permeates slowly (Figure 4E; Videos S8–S10, Supporting Information). This may be due to semi‐opening pores distributed inside. The hydrophilic component of DDRS shows obvious hydrophilicity, concurrently, the asymmetrical structure and hierarchical pore distribution make the two sides show different penetration effects.

**Figure 5:**
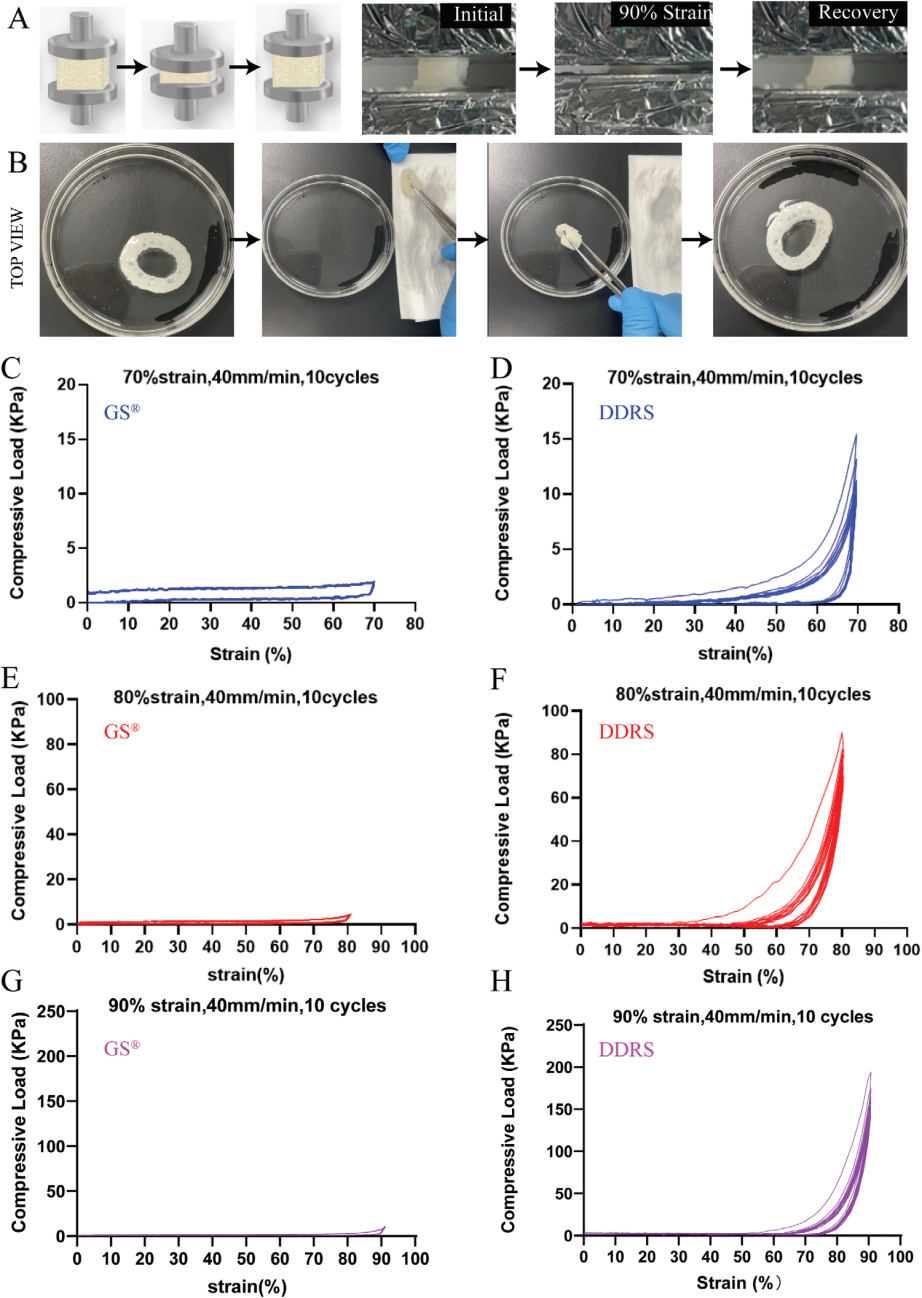
Compression elasticity and shape memory of material. A) Schematic diagram of compression test direction. And images of cyclic compression at 90% strain of DDRS. B) Shape recovery ability of DDRS in water (Video S11, Supporting Information). C,E,G) Under the speed of 40 mm min^−1^, the compression curve of the GS in ten cycles, and the strain was 70%, 80%, and 90%, respectively. D,F,H) Under the speed of 40 mm min^−1^, the compression curve of DDRS was ten cycles, and the strain was 70%, 80%, and 90%, respectively (Video S12, Supporting Information).

DDRS also showed excellent compressive elasticity. It remained intact for ten compression cycles at 90% strain (**Figure** **5**A). After squeezing out the water, the DDRS can also recover its original shape in water (Figure 5B; Video S11, Supporting Information), suggesting that DDRS has exceptional shape‐memory response in water.^[^ ^38^, ^39^ ^]^ Furthermore, compared with GS, DDRS exhibited significantly better mechanical strength under cyclic compression (Figure 5C–H; Video S12, Supporting Information). Under identical compression rate and strain (40 mm min^−1^ and ten cycles), the maximum compressive strength of the DDRS can be ten times higher than that of the GS. Especially at 90% strain, the maximum compressive strength of DDRS can reach 200 kPa, which is good to recover the irregular deep wound shape.^[^ ^40^, ^41^, ^42^, ^43^ ^]^

### 2.5 Degradation and Cytotoxicity of DDRS

**Figure 6:**
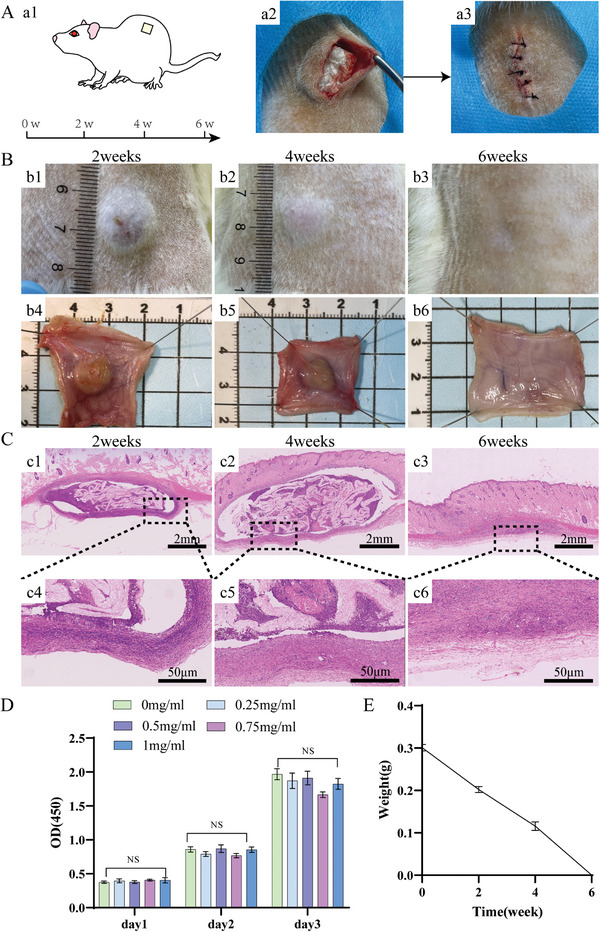
Histocompatibility, and degradability of DDRS. A) Schematic diagram of subcutaneous implantation test of DDRS in rats (a1), and the operative photos of subcutaneous insertion (a2, a3) (n = 3). B) Pictures of subcutaneous masses after subcutaneous implantation for 2 weeks (b1, b4), 4 weeks (b2, b5), and 6 weeks (b3, b6). C) HE staining pictures at different time points. D) DMEM soaked with DDRS for 24 h in the extract (the concentration is 1, 0.75, 0.5, and 0.25 mgmL^−1^), after 1, 2, 3 days of culture, the cell viability was tested by CCK‐8(n = 5). E) the in vivo degradation curve (n = 3).

Both degradability and biocompatibility are crucial for biomaterials. Therefore, we evaluated the degradability of the DDRS by subcutaneous implantation test in rats (**Figure** **6**A(a1)). We implanted the DDRS of 1 cm diameter into the back of rats (Figure 6A(a2,a3)) and subsequently measured size and weight at the 2nd, 4th, and 6th week. The remaining weight was significantly reduced by 2nd, 4th, and 6th week, the DDRS was degraded entirely (Figure 6B,E). We conducted an in vitro degradation experiment on DDRS by immersing it in simulated gastric juice. The results were shown in the Figure S1 (Supporting Information). It was showed that after 1 week of soaking in simulated gastric juice, ≈10% of the DDRS was degraded. Therefore, according to the results, we can estimate that the degradation time in vivo was ≈6 weeks, which is basically conforms to the clinical repair time of gastric perforation (≈1 to 2 months). The histological assessment revealed a reduction in foreign body reactions over time, as demonstrated by HE staining (Figure 6C). DDRS was soaked in a complete medium for 24 h to evaluate the toxic effect of DDR on cells. There is no significant difference between the saturated solution of each concentration sample and the control group, indicating that DDRS has no cytotoxicity (Figure 6D, n = 3). In addition, we performed a live‐dead cell staining experiment to assess the cytocompatibility of DDRS (Figure S2, Supporting Information), the results also shown that DDRS had good cytocompatibility. We also evaluated DDRS cytocompatibility by in vitro validation of cell adhesion growth assessment on DDRS. DDRS slices were incubated with NCM460‐SHNC cells labeled with green fluorescence, and cell growth was observed using a fluorescence microscope on day 1, 2, and 3, respectively. As shown in the Figure S3 (Supporting Information), it is evident that cells can thrive well on DDRS sheets at different time points. Moreover, with the passage of time, increasing fluorescence indicates an increase in the number of cells These results suggest that DDRS sheets are non‐toxic to cells and promote cell adhesion, growth, and proliferation.

### 2.6 Hemostatic Properties of DDRS

**Figure 7:**
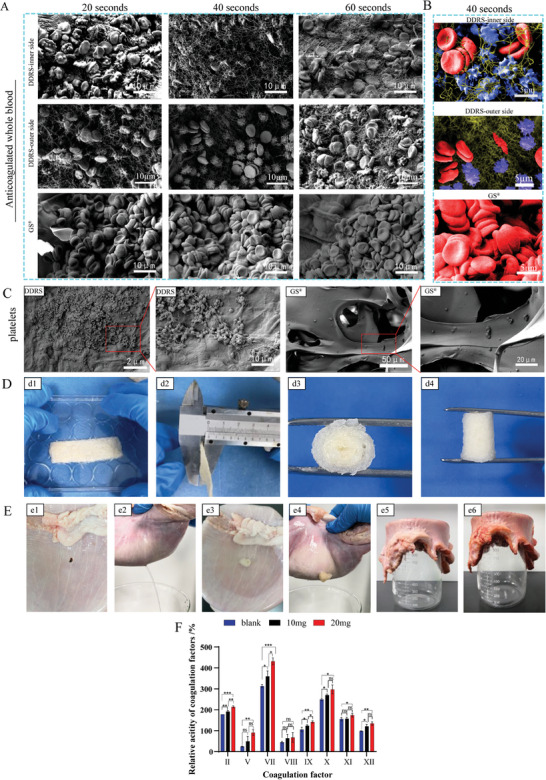
Hemostasis and repair of gastric perforation. A) SEM images of the inner and outer sides of DDRS after exposure to whole blood (sodium citrate) at different time points (20, 40, and 60 s). GS was used as the control group. B) SEM images after exposure to whole blood (sodium citrate) for 40 s. The blue area represents activated platelets, the red area is red blood cells, the yellow area represents the fibrin reticulum. C) SEM photos showing the adhesion of platelets on DDRS and GS. D) The preparation process of the DDRS occluder. Flatten the DDRS and then curl it along the long axis to get an onion roll‐like device (d1, d2). View pictures of the device from different perspectives (d3, d4). E) DDRS was used for sealing of a leaking in vitro pig stomach. A 5 mm wound was created on the pig stomach (e1), which caused a hole to leak (e2), DDRS was then employed to seal the gap(e3), following which the hole no longer leaked (e4). Put the pig stomach upon a beaker, pour the simulated gastric juice on the pig stomach, observe immediately (e5), and observe after 24 h(e6). F) The impact of DDRS on the activity of coagulation factors. (n = 3, mean ± SD, ^***^ *p* <0.001, ^**^ *p* <0.01, ^*^ *p* <0.05).

Platelet aggregation and fibrin network formation occurred after the 40 s of DDRS and blood (sodium citrate) contact, but these phenomena were not found GS group,^[44‐^ ^45^ ^]^ which served as a control (**Figure** **7**A,B). The platelets on the surface of the DDR significantly aggregated and formed a fibrin network after 1 min, while GS group, only minimal aggregated platelets were found (Figure 7C). The hemostatic ability exhibited by DDRS may due to its natural biomaterials based on chitin and polysaccharides. Current research shows that chitin has a hemostatic ability.^[^ ^46^, ^47^, ^48^ ^]^ At the same time, DDRS exhibits a biomimetic 3D microstructure and appearance, which benefits platelet adhesion and aggregation.^[^ ^47^ ^]^ We also evaluated the procoagulant effect of DDRS in vitro (in Figure S4, Supporting Information). The results exhibited that the procoagulant effect of DDRS was greater than that of GS, which are commonly used as hemostatic materials in clinical practice. DDRS is a biomaterial in contact with blood. Therefore, we evaluated the blood compatibility of DDRS in vitro (as shown in Figure S5, Supporting Information). After incubation with DDRS or GS for 1 and 8 h, the supernatant showed a light red color akin to that of physiological saline treatment (Figure S5A, Supporting Information). Statistical analysis revealed no significant difference between the DDRS and NS group at both 1 and 8 h, indicating that DDRS exhibits good blood compatibility (Figure S5B, Supporting Information). For the aim of investigating the interaction between DDRS and the coagulation system, an automatic coagulometer was employed to assess the impact of DDRS on coagulation factors. As presented in Figure 7F, DDRS showed an elevation in the activity of coagulation factors, with a notable increase in the activity of factor VII. In vivo hemostasis, we constructed a rat liver hemorrhage model and found that the hemostatic effect of DDRS (Figure S6, Supporting Information) was significantly better than that of the blank, and was resembling that of GS, which is a commonly used hemostatic material in clinic practice, indicating that DDRS had a good hemostatic effect in the in vivo liver hemorrhage model.

### 2.7 Sealing of a Leaking In Vitro Pig Stomach by DDRS Occluder

Due to the water absorption, compression elasticity, hemostatic ability, and different permeability on both sides, DDRS was used to repair gastric perforation.^[^ ^49^, ^50^, ^51^ ^]^ The DDRS was folded along its long axis to get an onion roll‐like device (Figure 7 D(d1,d2)). For in vitro sealing of a leaking pig stomach, a 5 mm wound was made on the pig stomach (Figure 7E(e1,e2)), and DDRS was used to seal the hole (Figure 7E(e3)) with a fast sealing (Figure 7E(e4)). It can seal the pig stomach filled with simulated gastric fluid (circular wound with a diameter of 5 mm) and no liquid leakage was found after 24 h (Figure 7 E(e5,e6)).

### 2.8 Enhanced Healing of Gastric Perforations in Pigs with Endoscopy Delivered DDRS

**Figure 8:**
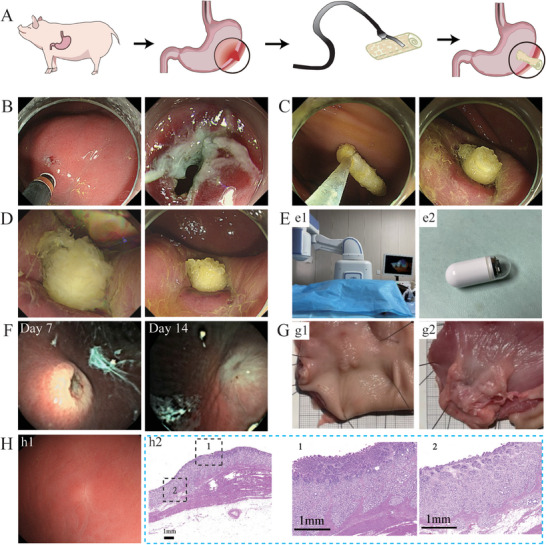
Evaluation of the closure and recovery of stomach perforations in pigs by endoscopic. A) Schematic diagram of treatment of gastric perforation pig. B) Create a perforation model of the stomach wall using an electric knife under endoscopic guidance. C) Put the material into the perforated part of the gastric wall (Video S13, Supporting Information). D) The material surface is sprayed with water to induce expansion, and the expanded material will seal the hole within 3 min. (Video S13, Supporting Information). E) Pictures of magnetic control bed (e1) and magnetic control capsule endoscopy (e2). F) Photos taken by magnetic capsule endoscopy after 7 days and 14 days of treatment. G) Specimens of perforated tissue of pig stomach. (g1) Mucosal side, (g2) Serous side. H) Pictures taken by endoscope 2 months after treatment and the healing and pathological results.

Endoscopic intervention can avoid injury caused by surgery.^[^ ^51^ ^]^ To demonstrate the feasibility of using endoscopic delivery to treat gastrointestinal perforation, we utilized an in vivo miniature porcine gastric perforation model (**Figure** **8**A). A 5 mm vertical perforation was made in the greater curvature of the stomach with electrocautery under the endoscope (Figure 8B). The device was clamped under the endoscope for sealing (Figure 8C; Video S13, Supporting Information). After 3 min of observation, it was found that the device gradually expanded and fixed in the perforated position (Figure 8D; Video S13, Supporting Information). On the 7th day, the device was wrapped by the gastric mucosa, the wound was gradually closed, and there was no fluid leakage around the wound. On the 14th day, the gastric mucosa at the surgical site was fully repaired (Figure 8F). Two months after treatment, gastric tissue was observed under endoscopy (Figure 8H), and the gastric mucosa at the surgical site was completely repaired. HE staining of the harvested gastric tissue showed that the closed gastric perforation was completely bridged with the regenerated mucosa, and the gastric mucosa achieved full‐thickness repair. The gastric mucosa at the repair site is thicker than the mucosa around the wound, which is a positive manifestation in the healing process of the gastric mucosa. The above results indicate that the DDRS device can be delivered to the perforation site by endoscope to release and promote wound closure and healing in vivo.

## 3 Conclusion

A multi‐stage porous natural hydrogel with super elasticity and surface characteristics derived from *Dictyophora rubrovalvata* is used as an internal wound repair device for treating gastric perforation. Minipig models of acute gastric perforation showed that the DDRS occluder prevented sealing of the perforation and promoted wound healing. At the same time, it is proved that this device can treat the perforation site through endoscopic delivery. Compared with the traditional methods for treating gastric perforation, the newly prepared hydrogel plugging device of natural origin has advantages, including simplified surgical procedures, easy operation, biocompatibility, and hemostatic effect, providing a new idea for the treatment of gastric perforation. In addition to the application of gastric perforation treatment, the application of our natural multi‐stage porous hydrogel in other fields needs further research.

## 4 Experimental Section

### Materials

*Dictyophora Rubrovalvata* (DR) was purchased from Yunnan, China. Gelatin sponges (GS) were purchased in a commercial market. The powder for sodium dodecyl sulfate (SDS) was purchased from Sangon Biotech Co., Ltd. located in Shanghai, China. Sodium fluorescein, cell count kit‐8 (CCK‐8), and Giemsa stain were procured from Beijing Solebo Technology Co., Calcofluor White Stain was purchased from Sigma. The rest of reagents were standard laboratory reagents. The animal studies conducted in this research adhered to the regulations outlined in the Guide for the Care and Use of Laboratory Animals, published by the US National Institutes of Health. Additionally, these studies were approved by both the Laboratory Animal Welfare and Ethics Committee of Third Military Medical University (AMUWEC20202115), and the Medical Ethics Committee of the Second Affiliated Hospital of Army Medical University, PLA (No.2023‐142‐01).

### Preparation of DDRS

The acellular technology to remove the excess tissue and retain the DRS is adapted from the whole organ perfusion acellular technology. The DRS were soaked in 0.1% SDS (sodium dodecyl sulfate) in deionized water for 3 days, changing the solution every 24 h, then washing it with sterile deionized water. Afterward, the decellularized DRS were stored overnight in a refrigerator at −80 °C and lyophilized for 24 h. The resulting DDRS can be tailored to any desired model, such as a circle or rectangle. By wetting and crimping DDRS and then drying it, an occluder was obtained.

### Microstructure and Ultra Microstructure and Porosity

The microstructures of DR and DDRS were examined by Micro‐CT and scanning electron microscope (SEM FEI/Phillips XL30). Micro‐CT images were processed by CTVOX software. The ultra microstructure of DR and DDRS was observed by section staining: the DR and DDRS was made into paraffin sections, and the structural characteristics of its inner, outer, and cross‐sections were observed by different staining H& E, PAS, Giemsa, and Calcofluor White Stain). In addition, the DDRS was made into ultra‐thin sections and used for transmission electron microscopy characterization.

### Establishment of a Mathematical Model for the Pore Structure of DRS

The cross‐section of fresh DRS was cut normal to the long axis into several segments. A digital camera was used to capture the cross‐sectional image of the DRS. After taking digital images, the diameters of all pores on the cross‐section were measured using ImageJ. The radius of the hollow circle inside the DRS was defined as R, and the average diameters of each layer of pores from inside to outside are D_1_, D_2_, and D_3_ (the average diameter is calculated as an arithmetic mean). The number of pores in each layer on the cross‐section were labeled as N_1_, N_2_, and N_3_ from the inside out. MATLAB software was used to analyze the correlation between parameters and to construct a mathematical model.

### Biomimetic DRS Model Printing

A 2.5 cm diameter DRS and solid silicone concentric cylinder models were obtained through 3D printing. Through a universal tensile tester (MTS E44.304), the cyclic compression tests of the two materials were carried out parallel and perpendicular to the transverse plane, respectively. In the direction parallel to the transverse plane, the compression speed is set to 200 mm min^−1^ and the compression strain is set to 80%. In the direction parallel to the transverse plane, set the compression speed to 200 mm min^−1^ and the compression strain to 80%. The stress–strain curves of each group were drawn after ten cyclic compressions.

### Mechanical Testing

The compression test was conducted on a universal tensile tester (MTS E44.304). The DDRS membranes and GS were prepared as discs with 1 cm diameter. After water absorption, the samples were compressed at a rate of 40 mm min^−1^ for ten cycles, and the compression strains were set at 50%, 70%, and 90%, respectively, to assess the compressibility and resilience of the specimens (n = 3).

### Water Absorption Behavior

DDRS square samples measuring 1 × 1 cm were weighed before water absorption (m_1_). The samples were then immersed in deionized water for different time points, and then removed the DDRS from the water and the weights were recorded (m_2_). The water absorption ratio is determined through the subsequent equation:

(8) $$\begin{array}{c} Waterabsorptionratio=\left(m_{2}-m_{1}\right)/m_{1} \end{array}$$

### Permeability Property

The DDRS samples measuring 1 × 2 cm was placed in a beaker containing safranine red stain solution (0.5% wt.%) with the outer side/inner side facing down, and the permeation process of the solution on different sides of DDRS was recorded by digital camera. DDRS samples were placed on the table with different faces down, and 50 µL Giemsa solution (0.5% wt.%) and sodium fluorescein solution (0.5% wt.%) were added to the surface of each group, respectively. A digital camera was used to record the penetration process of the solution on different sides of DDRS. When sodium fluorescein solution was added, UV light source was added to excite fluorescence. Similarly, put the DDRS samples on the clean supporting surface with the inner side/outside side down respectively, and the GS was used as the control group. 10 µL blood (sodium citrate) were added to each sample and blood absorption behavior of different materials with photographed.

### Shape‐Memory Property

DDRS membrane was compressed to extrude excess water and achieve a stable shape. Subsequently, the shape‐fixed DDRS membrane was exposed to water, and the shape recovery process was captured by a digital camera.

### Whole Blood Reaction and Platelet Adhesion Test

The whole blood and platelet adhesion on DDRS and GS samples were observed using scanning electron microscope (SEM). The whole blood was obtained from healthy human volunteers and anticoagulated with sodium citrate (3.8% wt.%) at a dilution ratio of 1:9 v/v (anticoagulant/blood). The samples were exposed to 100 µL whole blood or platelet suspension in a 24‐well microplate. Upon being exposed for varied durations, the specimens were rinsed with PBS, subsequently fixed with a 2.5% glutaraldehyde solution, and subject to dehydration via a succession of graded alcohol mixtures. Ultimately, the samples were dried. After drying, samples were sputter‐coated with gold before SEM imaging.

### In Vitro Hemolysis Test

Fresh anticoagulant blood (6 mL) (sodium citrate) was collected from rats and centrifuged (1500 rpm, 5 min) to isolate red blood cells. The red blood cells were then diluted to 10% v/v with physiological saline and divided into small vials of 500 µL each. 5 mg DDRS and GS were added to each vial and incubated with red blood cells at 37°C. The positive control group were treated with 0.1% Triton‐X, and the negative control group were treated with physiological saline. After incubation for 1  and 8 h, centrifuge (500xg) for 10 min and transfer the supernatant to a new 96‐well plate. To assess blood compatibility, the hemoglobin absorbance of each supernatant at 540 nm was measured. (n = 3)

### In Vivo Hemostasis Test

DDRS and GS were prepared into discs with diameter 8 mm and thick 4 mm. The anesthetized rats were opened to the abdomen with a scalpel to expose the liver. A quantitative filter paper with a diameter of 12.5 cm was placed under the hepatic lobe of rats, and then a round defect bleeding model was made in the liver by a perforator with a diameter of 4 mm. The prepared material is wetted with physiological saline and the excess water is removed. After stable bleeding of the liver, the material was put into the bleeding position, the hemostasis of each group was observed. From the beginning after the hemostatic material was put in, the filter paper was taken out and weighed after 3 min of observation, and the blood loss of each group was calculated. Bleeding without any treatment was used as a negative control (n = 3).

### Examination of the Effect on Coagulation Components

Platelet‐rich plasma (PRP) is acquired from healthy human volunteers, and coagulation activity is accomplished by utilizing an automated coagulation device (CS‐5100). The coagulation factor reagents had been previously formulated in the automatic coagulant machine prior to the trial. After frozen with liquid nitrogen, the DDRS was pulverized into a fine powder, and 1 mL of PRP was mixed with either 10 or 20 mg of DDRS powder. These mixtures were then separately placed for 10 min and subsequently. Centrifuged at a speed of 3500 rpm for 15 min. The supernatant (300 µL) was taken into the test cup for analysis, and the activity of coagulation factors was recorded. Physiological saline was used as a control in this study.

### Cytocompatibility Test

CCK‐8 and live/dead staining evaluated the cytocompatibility of DDRS. The DDRS, after sterilization, was immersed in PBS for washing. The samples were then incubated in a complete medium for 24 h. 100 µL of extracts (0.25, 0.5, 0.75, and 1 mg mL^−1^) were added to 3T3 fibroblast suspension (5 × 10^3^/well) in 96 well plates. Complete medium as blank control group. After culturing cells at 37 °C for 1, 2, and 3 days, CCK‐8 reagent was added to each well and incubated for another 40 min. The optical density was then determined using a microplate reader at 450 nm. And the cell growth was observed after staining with AO/PI staining solution.

### In Vitro Validation of Cell Adhesion Growth Assessment on DDRS

DDRS samples (0.5 × 0.5 cm) were incubated with NCM460‐shnc cells (5 × 10^3^/well) labeled with green fluorescence, and cell growth was observed using a fluorescence microscope on day 1, 2, and 3, respectively.

### In Vivo Degradation Studies

DDRS membranes were cut into 1 × 1 cm and sterilized using cobalt‐60 irradiation. After the intraperitoneal injection of 0.3 mL pentobarbital sodium (3 wt.%) to 8 weeks old male specific‐pathogen free (SPF) Spragu‐Dawleys (SD) rats, the hairs on both sides of the back of the rats were shaved. About 1 cm incisions were made using a scalpel on one side of the rat's back and the materials were placed under the skin at one side of the incision. The incision was closed by suturing. Digital photos were taken at the 2nd, 4th, and 6th week to monitor the skin condition of the DDRS. These photos were taken as a means of visual observation and analysis of the skin's appearance and any changes that may have occurred over time. The animals were sacrificed and the thickness and area of the harvested DDRS measured. The remaining tissue was fixed in 10% paraformaldehyde, embedded in paraffin, sectioned, and stained for HE for histological observation of material degradation and inflammatory cells infiltration and proliferation of in the tissue around the material.

### In Vitro Degradation Studies

The DDRS was cut into 5.5 mg weight samples and placed in a 24‐well plate, Each wall was added with 2 mL of simulated gastric juice, and then incubated at 37 °C. The samples were retrieved on day 1, 3, 5, and 7 respectively, then subjected to vacuum freeze‐drying and weighing (n = 4).

### In Vitro Gastric Perforation Closure Pig Stomach

The DDRS was cut into a rectangle, wetted with pure water, curled into an onion‐like device from the outer side, and dried. Pig stomach purchased from a market was placed on the rim of a beaker where the serous membrane facing up. A 0.5 cm long cut was made on the large radian side of the stomach and were sealed with DDRS. 50 mL simulated gastric fluid was poured on the sealed surface to evaluate any leakage within 24 h.

### Endoscopic Closure of Porcine Gastric Perforation Model with DDRS Occluder

Pigs weighing ≈15 kg were given anesthesia and positioned on the surgical table. The endoscope was inserted into the pig's stomach and an electric knife was used to form a hole ≈5 mm in the stomach wall under the endoscope. The excess tissue was removed with biopsy forceps to form an artificial model of gastric perforation. The prepared DDRS occluder was dried at room temperature for 24 h. Clamp the DDRS occluder with the forceps in front of the endoscope and transport it to the wound through the esophagus to prevent perforation. Three minutes after operation, the DDRS occluder was sprayed with water to dilate and observe the perforation. Wound healing was evaluated by magnetic capsule endoscopy on the 7th and 14th day after operation. After euthanasia, the perforated tissue was removed, treated with formalin (10% solution) for 24 h, the specimens were preserved in paraffin for subsequent histological analysis.

### Statistical Analysis

SPSS23.0 software was used for data statistical analysis. The measurement data is expressed as mean ± SD, and the measurement data between multiple groups are analyzed using one‐way ANOVA. The difference represented by *p* <0.05 is statistically significant.

## Conflict of Interest

The authors declare no conflict of interest.

## Supporting information

Supporting Information

Supplemental Video 1

Supplemental Video 2

Supplemental Video 3

Supplemental Video 4

Supplemental Video 5

Supplemental Video 6

Supplemental Video 7

Supplemental Video 8

Supplemental Video 9

Supplemental Video 10

Supplemental Video 11

Supplemental Video 12

Supplemental Video 13

## Data Availability

The data that support the findings of this study are available from the corresponding author upon reasonable request.
