# Supplementary material for: Analyzing Mushroom Structural Patterns of a Highly Compressible and Expandable Hemostatic Foam for Gastric Perforation Repair
Source: Adv Sci (Weinh). 2024 Mar 4;11(21):2306917. doi: 10.1002/advs.202306917 (PMC11151031; doi:10.1002/advs.202306917)
Supplement: Supplementary file 1 — Supporting Information [file ADVS-11-2306917-s010.pdf]

ADVANCED SCIENCE
Open Access

Supporting Information

for *Adv. Sci.*, DOI 10.1002/advs.202306917

Analyzing Mushroom Structural Patterns of a Highly Compressible and Expandable Hemostatic Foam for Gastric Perforation Repair

Zhenzhen Shu, En Liu, Yu Huang, Qiang Luo, Tongchuan Wang, Xin Li, Kibret Mequanint, Shiming Yang, Malcolm Xing* and Chaoqiang Fan**

Supporting Information

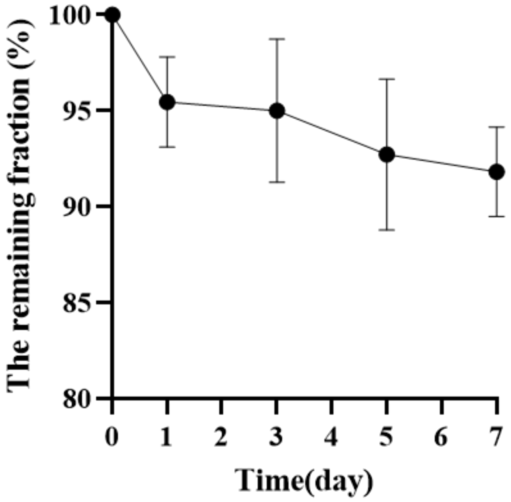

Figure S1. Degradation curve of DDRS in simulated gastric juice. (n=4)

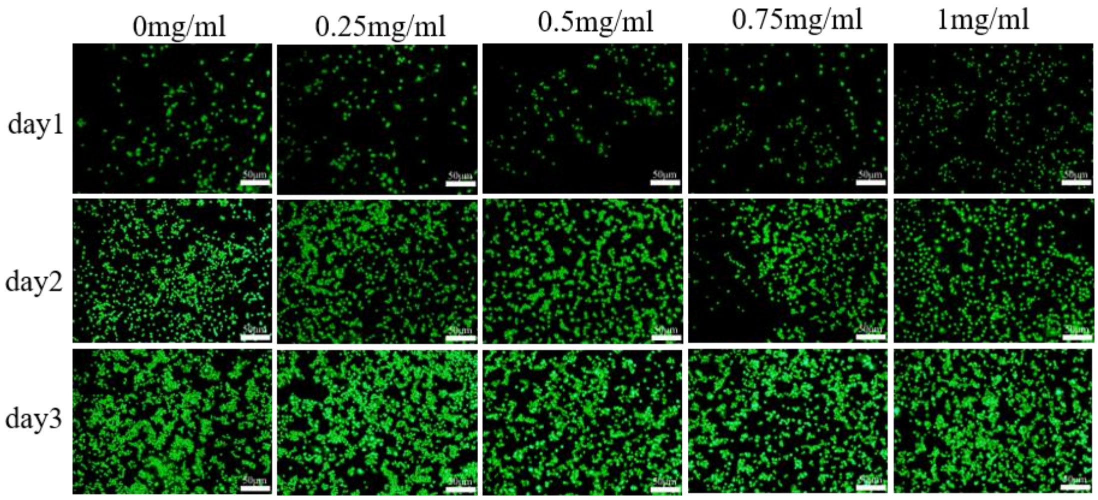

Figure S2. Live-dead cell staining images. 3T3 cells were incubated with DDRS extracts of different concentrations at different time points (day 1, day 2, day 3), and the cell growth was observed after staining with AO/PI staining solution. (The scale bar is 50 μm)

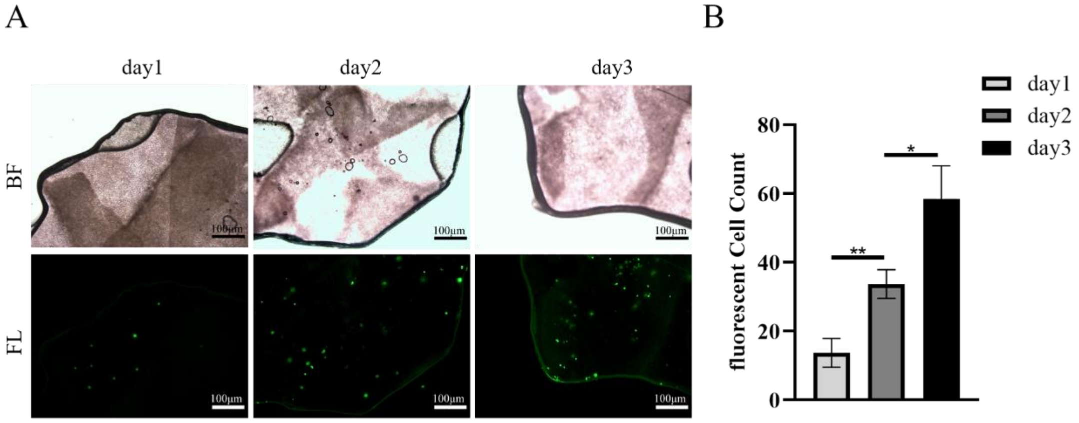

Figure S3. Evaluation of cell adhesion and growth *in vitro*. (A)DDRS and NCM460-shnc cells were incubated together, and the cell growth was observed by fluorescence microscopy on day 1, 2 and 3, respectively. (BF represents the bright field under normal light, FL represents the fluorescence microscope, and NCM460-shnc cells show green fluorescence under FL.) (B) Fluorescent Cell Count at Different Time Points. (**P*<0.05, ***P*<0.01, n=3)

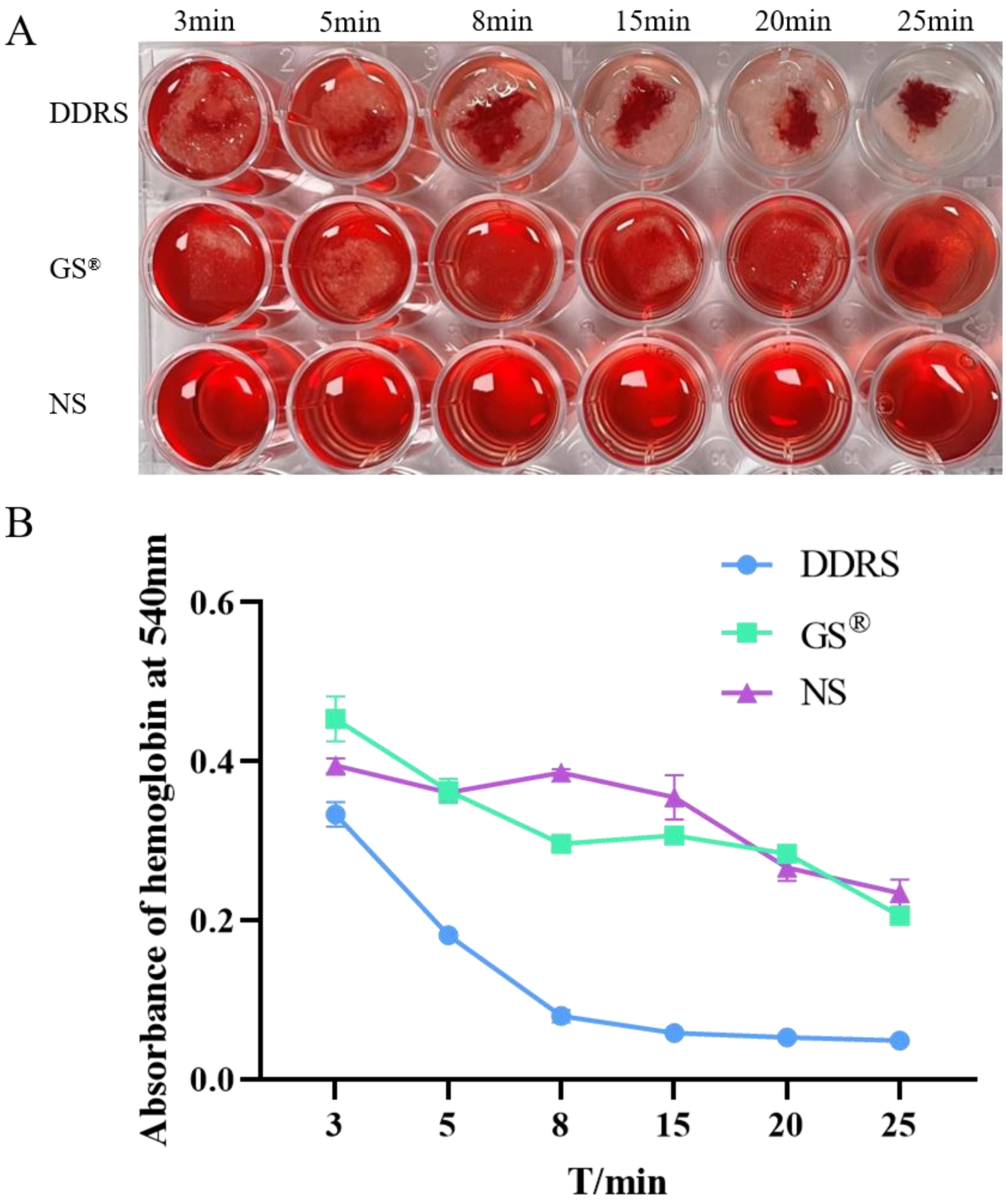

Figure S4. Hemostatic test *in vitro*. (A) Picture of whole blood coagulation test: DDRS reacted with anticoagulant whole blood (sodium citrate) for different time, then rinsed with pure water; GS® (gelatin sponge) as control group, NS (normal saline) as negative control; (B) whole blood coagulation test curve: the supernatant after washing in each group was placed on 96-well plate, and the absorbance of hemoglobin at 540 nm was measured.

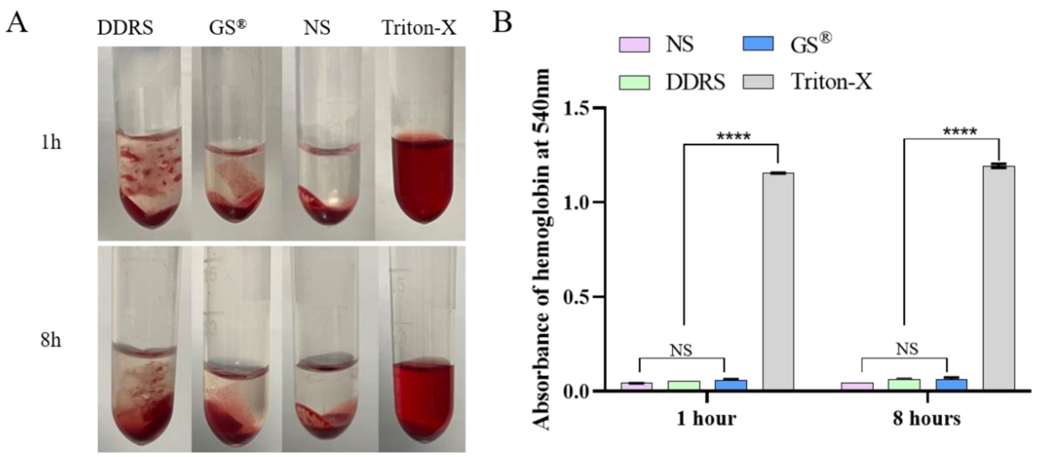

Figure S5. Blood compatibility test. (A) DDRS and GS® were co-incubated with rat red blood cells (10% v/v) diluted with normal saline at 37 ℃. 0.1% Triton-X and saline were used as positive control group and negative control group. After 1 hour and 8 hours incubations, (500 × g) centrifugal 10 minutes was performed, and the pictures of each group were taken. (B) transfer the supernatant to the new 96-well plate. The absorbance of hemoglobin at 540 nm was determined. (****P<0.0001, n=3)

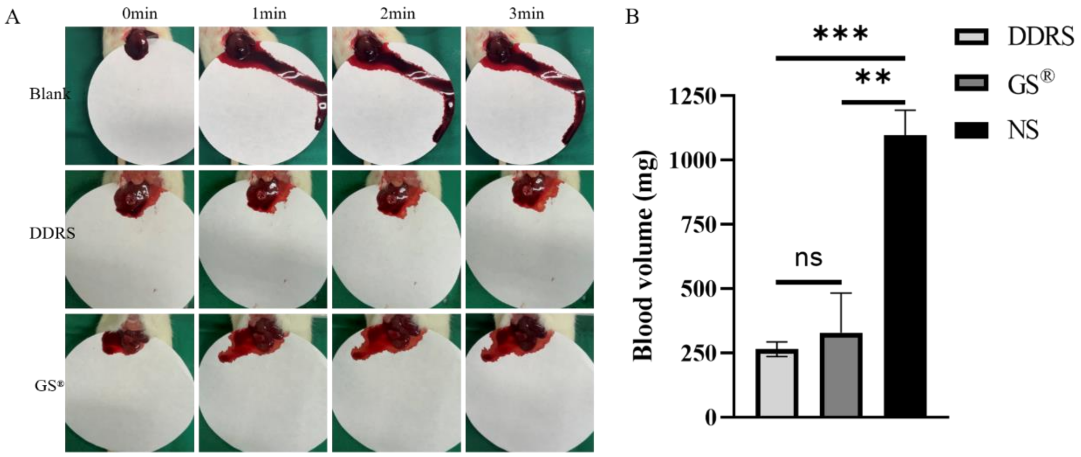

Figure S6. Hemostatic test *in vivo*. (A) Rat liver hemostasis test. NS stands for saline and GS® stands for gelatin sponge. (B) Statistics of bleeding volume in each group. (****P*<0.01, ****P*<0.001, n=3)
